# Widely-targeted metabolomics and transcriptomics identify metabolites associated with flowering regulation of Choy Sum

**DOI:** 10.1038/s41598-024-60801-4

**Published:** 2024-05-09

**Authors:** Xinmin Huang, Yunna Zhu, Wei Su, Shiwei Song, Riyuan Chen

**Affiliations:** 1 Guangdong Provincial Engineering Technology Research Center for Protected Horticulture, College of Horticulture, South China Agricultural University, Guangzhou, 510642 Guangdong People’s Republic of China; 2 College of Biology and Food Engineering, Guangdong University of Petrochemical Technology, Maoming, 525000 Guangdong People’s Republic of China

**Keywords:** Plant development, Plant physiology

## Abstract

Choy Sum, a stalk vegetable highly valued in East and Southeast Asia, is characterized by its rich flavor and nutritional profile. Metabolite accumulation is a key factor in Choy Sum stalk development; however, no research has focused on metabolic changes during the development of Choy Sum, especially in shoot tip metabolites, and their effects on growth and flowering. Therefore, in the present study, we used a widely targeted metabolomic approach to analyze metabolites in Choy Sum stalks at the seedling (S1), bolting (S3), and flowering (S5) stages. In total, we identified 493 metabolites in 31 chemical categories across all three developmental stages. We found that the levels of most carbohydrates and amino acids increased during stalk development and peaked at S5. Moreover, the accumulation of amino acids and their metabolites was closely related to G6P, whereas the expression of flowering genes was closely related to the content of T6P, which may promote flowering by upregulating the expressions of *BcSOC1*, *BcAP1*, and *BcSPL5*. The results of this study contribute to our understanding of the relationship between the accumulation of stem tip substances during development and flowering and of the regulatory mechanisms of stalk development in Choy Sum and other related species.

## Introduction

Flowering Chinese cabbage (*Brassica campestris* L. ssp. *chinensis* var. utilis Tsen et Lee), also known as Choy Sum or cai xin, is a subspecies of Chinese cabbage that is widely grown in commercial production fields and home gardens in East and Southeast Asia^1,2^. Choy Sum is a typical stalk vegetable, whose stalk development plays a pivotal role in the quality and yield of the crop. Stalk development in Choy Sum includes processes, such as stem elongation, thickening, and flowering, which are subject to complex regulation^2^.

Choy Sum is unique from other cabbage species as it does not require vernalization for flowering and bolting. However, studies have shown that its bolting and flowering are affected by low temperatures^2,3^. Similarly, our previous research indicated that gibberellin (GA) and low-temperature conditions can promote the flowering and bolting of Choy Sum^2,3^. Moreover, the application of indole-3-acetic acid (IAA) can increase endogenous GA and IAA content in Chinese cabbage, promote cell growth, and induce stem elongation in Choy Sum^4^. Notably, GA can upregulate the expression of the *BcSOC1* gene, a member of the *DELLA* gene family that encodes DELLA proteins, which negatively regulate GA signal transduction; its overexpression can promote flowering^5,6^. The BcRGL1-mediated gibberellin signaling pathway plays an important role in the early bud differentiation process of different cabbage varieties, thereby affecting bolting and flowering^5^. Knock out of *BcSOC1* in Choy Sum delays flowering, and gene expression analyses have indicated that *BcSOC1* can promote stem cell growth by upregulating genes, such as *EXH*, and that the protein BcSOC1 can interact with BcAGL6 and BcAGL24 to promote flowering^6^. In Choy Sum, BcKNOX1 may regulate the effects of GA on bolting and flowering by interacting with BcRGA1 and BcRGL1^7^. In cauliflower stems, BcRGA1 also interacts with BcNF-YA8, BcNF-YB14, BcNF-YB20, and BcNF-YC5 during the GA-mediated development of the stems^8^. These findings indicate that *BcSOC1* is a key flowering gene in Choy Sum.

Metabolite accumulation, such as that of carbohydrates, within stem tissue is the basis for stem growth and flowering, playing a pivotal role in establishing the nutritional value of the stem. Carbohydrates are essential metabolic products that provide energy for plant growth, serve as important components of plant cells, and serve as signaling molecules for regulating plant growth and development^9^. Moreover, carbohydrates stored in tissues can participate in the regulation of other key metabolic pathways for plant growth through synthesis and decomposition, including the synthesis of amino acids, proteins, membrane lipids, and cell wall components^10^. As autotrophic organisms, plant leaves synthesize sugars through photosynthesis, which are then transported to the cells of all other organs, including the roots, stems, seeds, and fruits^9,11^. The transmembrane transport of sugars is mediated by sugar transport proteins, comprising the monosaccharide transporter (MST), sucrose transporter carrier (SUT/SUC), and sugar-will-eventually-be transported (SWEET) families^12–14^. These sugar transporters control the distribution of sugars in different plant tissues by responding to internal and external signals, thereby regulating plant growth and development^14^. Despite the pivotal role of metabolite accumulation in Choy Sum stalk development, no research has investigated the metabolic changes during its development, especially in shoot tip metabolites.

Glucose, an important class of monosaccharides, produces-6-phosphate (G6P) under the action of hexokinase (HXK). This important step serves as the control center for plant metabolic pathways, wherein important compounds such as amino acids, fatty acids, and starch can be produced. Many other secondary metabolites are also synthesized during this step, including hormones, pigments, and cell wall polymers^15,16^. For example, trehalose is a nonreducing disaccharide composed of two glucose moieties. Trehalose-6-phosphate (T6P) is an important sucrose metabolism signaling metabolite that regulates plant carbon assimilation and sugar status, connecting plant growth and development to metabolic status^16–18^. In Arabidopsis, the overexpression of *AtTPS1*, which encodes the T6P synthase (TPS), leads to the accumulation of T6P and promotes flowering. In contrast, the overexpression of *AtTPP*, which encodes the enzyme trehalose-6-phosphate phosphatase, reduces T6P content and inhibits flowering^19^. Therefore, a close correlation exists between T6P and plant flowering regulation.

Currently, research on Choy Sum has mainly focused on the regulation of flowering and stem growth^2,6,20,21^. However, no studies have focused on the metabolic changes during Choy Sumit development, especially the changes in shoot tip metabolites and their relationship with growth and flowering. Therefore, the present study, using a widely targeted metabolomics approach, aimed to analyze the changes in shoot tip metabolites during the developmental stages of Choy Sum stems seedling (S1), bolting (S3), and flowering (S5). We also compared the results of the metabolomic analysis with existing transcriptome data^2^. This comprehensive approach aims to explore the relationship between cabbage metabolites and cabbage growth and flowering, providing new insights into the regulatory mechanisms of cabbage stalk growth and development.

## Results

### Overview of the metabolic profiles of Choy Sum stalk at three development stages

The reproducibility of the experiment was analyzed using quality control samples, and the results show that the positive-ion TIC (Fig. S1A) and negative-ion TIC (Fig. S1B) diagrams of the different QC samples overlapped, indicating that the metabolite extraction and determination were accurate and repeatable. In total, 493 metabolites were identified in the Choy Sum stalk samples. Among these, 271 and 222 were detected in positive and negative-ion modes, respectively (Table S1). The metabolites were classified into 39 categories, including organic acids, amino acids and derivatives, nucleotides and derivatives, hydroxycinnamoyl derivatives, lipids, glycerophospholipids, flavones, quinates and derivatives, flavonols, lipids, fatty acids, carbohydrates, flavone C-glycosides, phenolamides, phytohormones, and vitamins. Within these, organic acids (11.34%), amino acid derivatives (9.74%), nucleotides and their derivatives (9.53%), and amino acids (5.68%) were the most abundant (Table 1).

**Table 1:** Total metabolites at different developmental stages of Choy Sum stalks.

Type	Number	Type	Number
All	493 (100%)	Vitamins	13 (2.64%)
Organic acids	56 (11.34%)	Anthocyanins	12 (2.43%)
Amino acid derivatives	48 (9.74%)	Lipids_Glycerolipids	12 (2.43%)
Nucleotide and its derivates	47 (9.53%)	Coumarins	11 (2.23%)
Amino acids	28 (5.68%)	Indole derivatives	10 (2.03%)
Hydroxycinnamoyl derivatives	26 (5.27%)	Benzoic acid derivatives	9 (1.83%)
Lipids_glycerophospholipids	26 (5.27%)	Flavanone	9 (1.83%)
Others	26 (5.27%)	Cholines	7 (1.42%)
Flavone	23 (4.67%)	Tryptamine derivatives	5 (1.01%)
Quinate and its derivatives	18 (3.65%)	Alcohols and polyols	4 (0.81%)
Flavonol	16 (3.25%)	Alkaloids	4 (0.81%)
Lipids_fatty acids	16 (3.25%)	Nicotinic acid derivatives	4 (0.81%)
Carbohydrates	14 (2.84%)	Catechin derivatives	3 (0.61%)
Flavone C-glycosides	14 (2.84%)	Isoflavone	2 (0.41%)
Phenolamides	13 (2.64%)	Pyridine derivatives	2 (0.41%)
Phytohormones	13 (2.64%)	Terpenoids	2 (0.41%)

**Figure 1:**
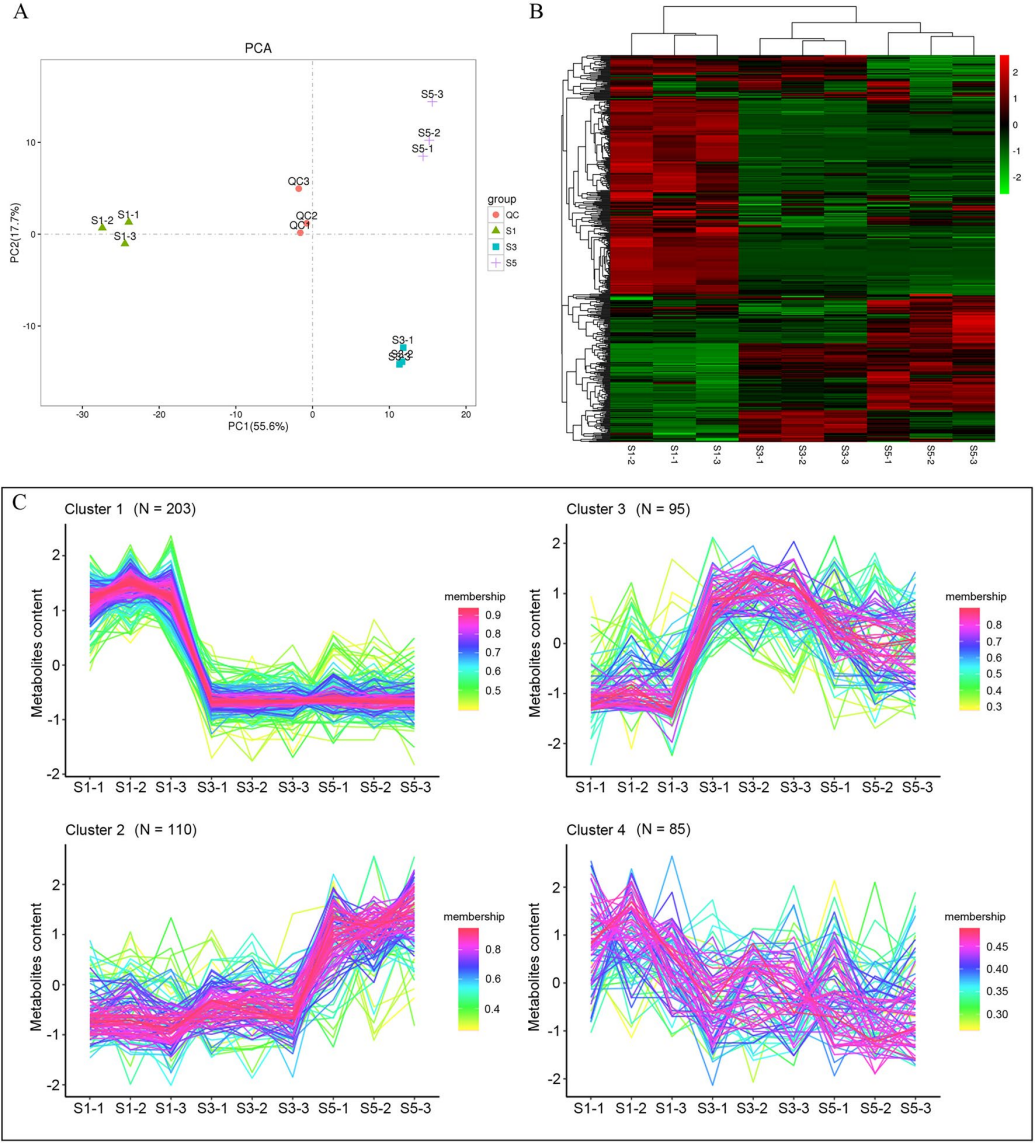
Multivariate statistical analysis of metabolites at different Choy Sum stalk developmental stages. (**A**) Pearson’s correlation coefficients of quality control (QC) samples and samples from all three developmental stages. (**B**) Hierarchical cluster analysis of samples from all three developmental stages. Values represent Z-scores for metabolite content data. (**C**) Trend cluster for metabolite content changes for samples from all three developmental stages.

To determine the changes in metabolites during the flowering stage of Choy Sum stalk development, we subjected the metabolomic data from the three main growth stages to a multivariate statistical analysis. Principal component analysis (PCA) revealed a clear separation between the developmental stages. The total variance was 73.3%, of which 55.6% was explained by PCA1 and 17.7% by PCA2, indicating that the metabolite profile changes dynamically during stalk development in Choy Sum (Fig. 1A). Moreover, S1 was distinctly clustered on the negative side of PC1, separate from S3 and S5, whereas samples S3 and S5 were grouped under PC2. A hierarchical cluster analysis (HCA) clustered S1 separately from S3 and S5 (Fig. 1B). Notably, numerous metabolites in Choy Sum stalks were altered during S1. All biological replicates across development stages were clustered together in both the PCA and HCA heatmaps, indicating that the metabolic profiling data were reliable (Fig. 1A,B).

Analysis using the TCseq package in R (v. 3.6.2) clustered the metabolite trends observed across the three developmental stages into four groups (Fig. 1C). Cluster 1 included 203 metabolites that were the most abundant in S1, but rapidly declined in S3 and S5. These metabolites included lipids, glycerophospholipids (12.32%), quinates and their derivatives (8.87%), nucleotides and their derivatives (8.37%), amino acid derivatives (8.37%), organic acids (6.9%), and flavones (5.42%) (Fig. S2). In cluster 2, 3,071 metabolites, including amino acids (17.27%), organic acids (15.45%), amino acid derivatives (13.64%), carbohydrates (6.36%), and flavones (6.36%), were the most abundant in S5 (Fig. S2). In cluster 3, 95 metabolites, including hydroxycinnamoyl derivatives (13.68%), nucleotide derivatives (13.68%), and amino acid derivatives (9.47%), were the most abundant in S3 (Fig. S2). In cluster 4, the levels of 85 metabolites stages, including organic acids (16.47%), lipid fatty acids (10.59%), others (10.59%), and amino acid derivatives (8.24%), gradually declined during the three developmental (Fig. S2).

### Identification of differentially accumulated metabolites at various Choy Sum stalk developmental stages

**Figure 2:**
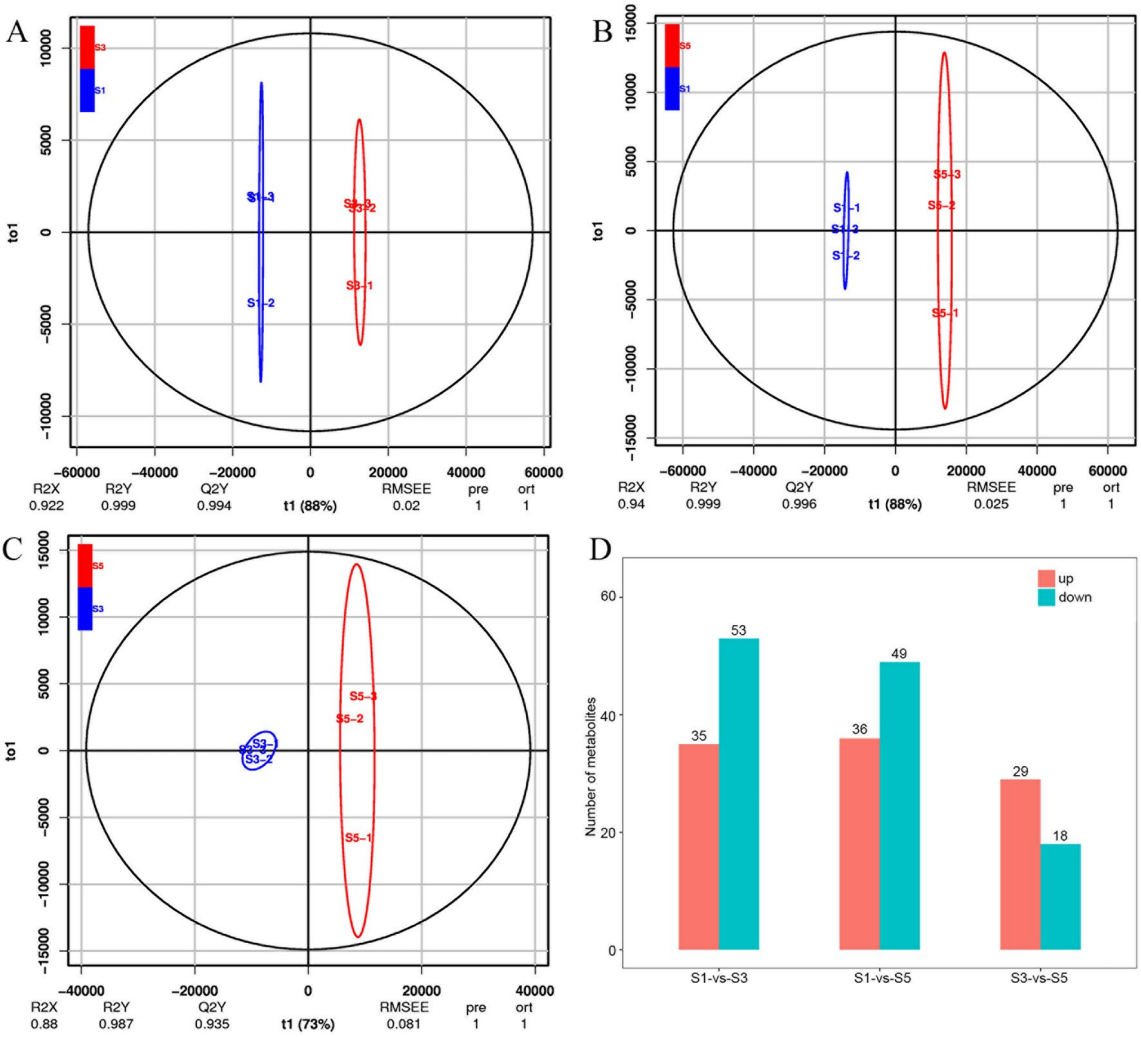
Orthogonal partial least-squares discriminant analysis (OPLS-DA) score (**A**: S1 vs. S3, **B**: S1 vs. S5, **C**: S3 vs. S5) and various metabolites identified in different pairwise comparisons of developmental stages. (**D**) Numbers of upregulated and downregulated metabolites in each stage pair.

To screen for key differential metabolites (DMs) at various Choy Sum stalk developmental stages, we conducted a supervised orthogonal projection to latent structure-discriminant analysis (OPLS-DA), constructed classification models, and discriminated various metabolic compositions among the growth stages. The OPLS-DA score plots (Fig. 2A–C) exhibited clear metabolic differentiation between S1 and S3, S1 and S5, and S3 and S5. R2Y and Q2 were > 0.90 for all three OPLS-DA score plots, indicating that the model is reliable and has strong predictive power. We verified the OPLS-DA model using cross-validation and 200 permutation tests (Fig. S3).

We set the variable importance in projection (VIP) ≥ 0.7 for the OPLS-DA model, performed a univariate statistical analysis *t*-test (p < 0.05), and identified 111 DMs from the three pairwise comparisons (Fig. 2D). Among these, 35 metabolites in S1 versus S3 were upregulated during S3, 53 metabolites in S1 versus S3 were downregulated during S3, 36 metabolites in S1 versus S5 were upregulated during S5, 49 metabolites in S3 versus S5 were downregulated during S5, 29 metabolites in S3 versus S5 were upregulated during S5, and 18 metabolites in S3 versus S5 were downregulated during S5.

**Figure 3:**
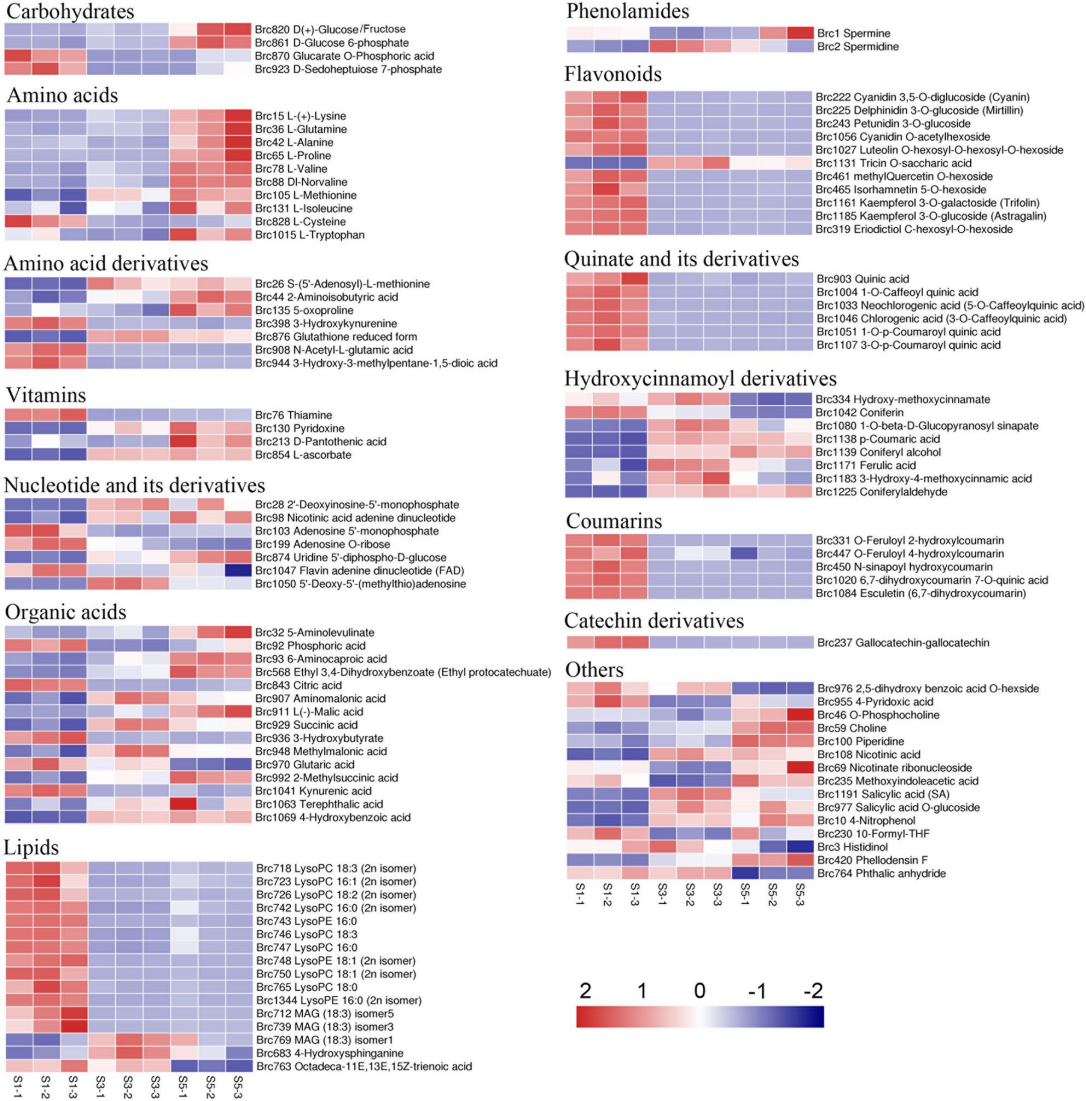
Heatmap of key significantly differential metabolites (DMs). Values are Z-scored for metabolite content.

Subsequent analysis revealed that the key DMs included carbohydrates (4), amino acids (10), amino acid derivatives (7), vitamins (4), nucleotides and derivatives (7), organic acids (15), lipids (16), phenolamides (2), flavonoids (11), quinates and derivatives (6), hydroxycinnamoyl derivatives (8), coumarins (5), catechin derivatives (1), and other metabolites (15) (Fig. 3). Among these, carbohydrates, such as glucose, fructose, and glucose 6-phosphate, continuously accumulated during stalk development and peaked at S5. Nine of the ten differentially expressed amino acids—l-( +)-lysine, l-glutamine, l-alanine, l-proline, l-valine, valine, l-methionine, l-isoleucine, and l-tryptophan—continuously accumulated during stalk development and peaked by S5. Vitamins B6, B5, and C continuously accumulated during stalk development and peaked by the S5 stage, whereas vitamin B1 levels decreased over time. In addition, eleven organic acids accumulated continuously during stalk development, with the levels of five organic acids ultimately decreasing. Most lipid substances decreased continuously during stalk development. Moreover, the contents of flavonoids, quinates, derivatives, coumarins, and catechin derivatives decreased during stalk development. In contrast, hydroxycinnamyl derivatives 1-O-beta-D-glucopyranosyl sinapate, p-coumaric acid, coniferyl alcohol, ferulic acid, and 3-hydroxy-4-methoxycinnamic acid continuously accumulated during stalk development.

### Transcriptome and metabolome correlation analysis in carbohydrate transport and metabolism

**Figure 4:**
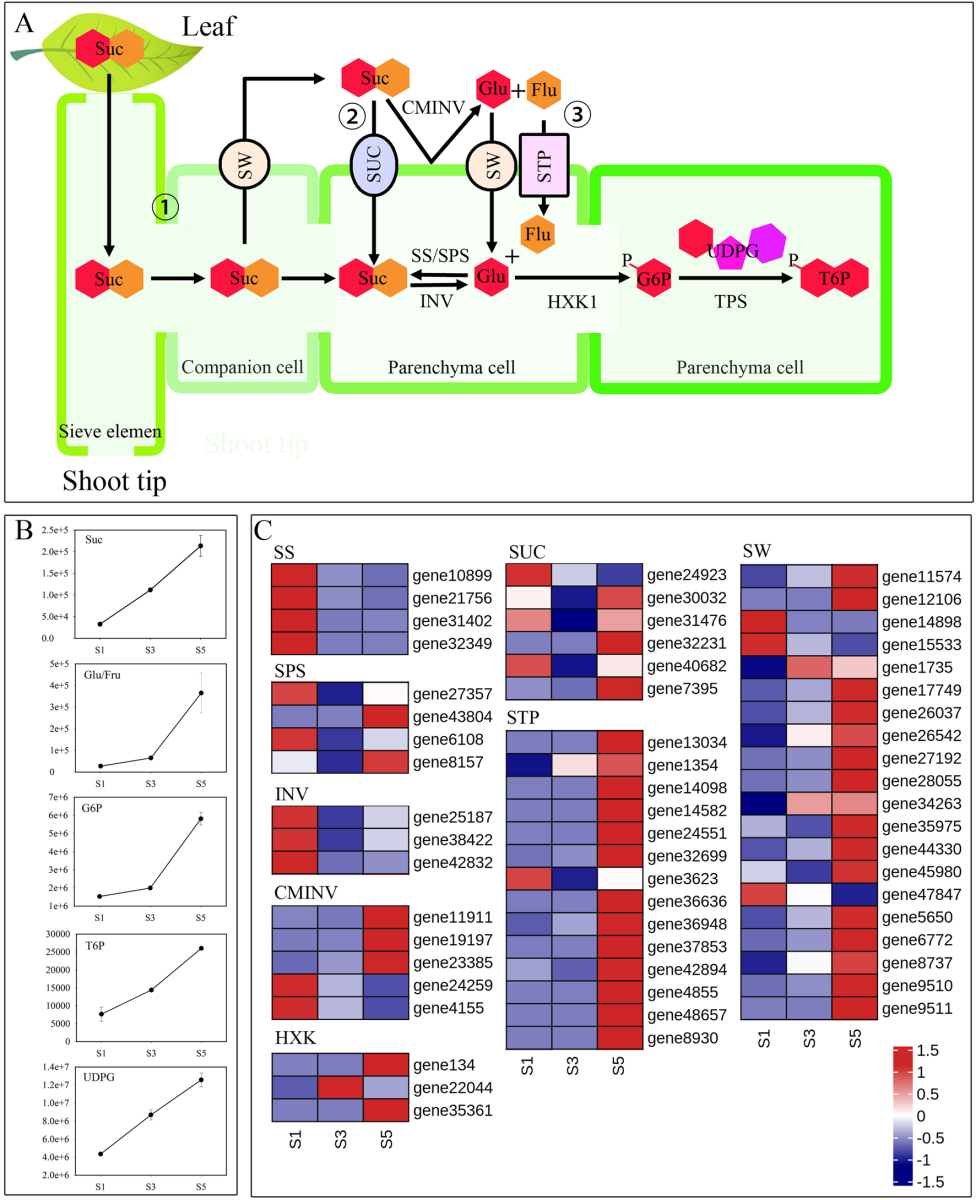
Schematic diagram of sucrose transport in Choy Sum stalk. (**A**) Diagram of sugar transport and metabolism at the shoot tip of Choy Sum stalk; (**B**) sucrose, glucose/fructose, trehalose 6-phosphate, glucose 6-phosphate, and uridine 5′-diphospho-d-glucose contents in samples. (**C**) Heatmap of sugar transport and metabolism gene expression. The line chart represents material change while the heatmap shows gene expression trends, values represent Z-scores for fragments per kilobase of transcript per million mapped reads (FPKM) data. *SW* sugar-will-eventually-be-exported transporter, *STP* sugar transport protein, *SUC* sucrose transport protein, *CWINV* cell wall invertases, *SS* sucrose synthase, *SPS* sucrose phosphate synthase, *INV* acid beta-fructofuranosidase, *HXK* hexokinase.

The trend analysis revealed that the levels of most carbohydrates were the highest at S5, including those of sucrose, glucose, fructose, G6P, and T6P (Figs. 3, 4A). Kyoto Encyclopedia of Genes and Genomes (KEGG) enrichment analysis indicated that differentially expressed genes (DEGs) were significantly enriched in “starch and sucrose metabolism” (ko00500)^7^. Of these, sucrose synthetase (SS) and sucrose phosphate synthetase (SPS) are key enzymes in sucrose biosynthesis. All four differentially expressed *BcSS* genes were downregulated during stalk development (Table S2), whereas only two of the four differentially expressed *BcSPS* genes were downregulated (Table S2). Of these, β-fructofuranosidases, such as vacuolar acid invertase (INV) and cell wall invertase (CWINV), convert sucrose into glucose and fructose. Moreover, during stalk development, three differentially expressed *BcINV* genes were downregulated (Table S2), whereas three of the five differentially expressed *BcCWINV* genes were upregulated (Table S2 and Fig. 4B). Three *BcHXK* genes were differentially expressed during stalk development, of which two were upregulated (Table S2, Fig. 4C). We subsequently analyzed the expression of genes that regulate sugar transporters during stalk development and found that 20 *BcSWEET*, 14 *BcSTP*, and 6 *BcSUC* genes were differentially expressed during stalk development. Of these, 16 *BcSWEET*, 13 *BcSTP*, and 3 *BcSUC* were upregulated (Fig. 4C).

To identify the regulatory network of carbohydrate transport and metabolism in Choy Sum stalks, we performed correlation tests between quantitative changes in sucrose, glucose, fructose, G6P, T6P, and transcripts at the three different developmental stages (Table S3). Based on the DEGs and DMs, 1 *BcSPS*, 5 *BcCWINV*, 2 *BcHXK*, 13 *BcSTP*, 2 *BcSUC*, and 12 *BcSWEET* genes exhibited high correlation coefficient values (r > 0.8 or r <  − 0.8) with carbohydrate accumulation (Table S3). Among these significantly correlated genes, gene23385 encoding *BcCWINV5*, gene4155 encoding *BcCWINV1*, and gene24923 encoding s *BcSUC4*, were positively correlated with carbohydrate accumulation (Table S3).

### Analysis of the association between G6P and other metabolites

**Figure 5:**
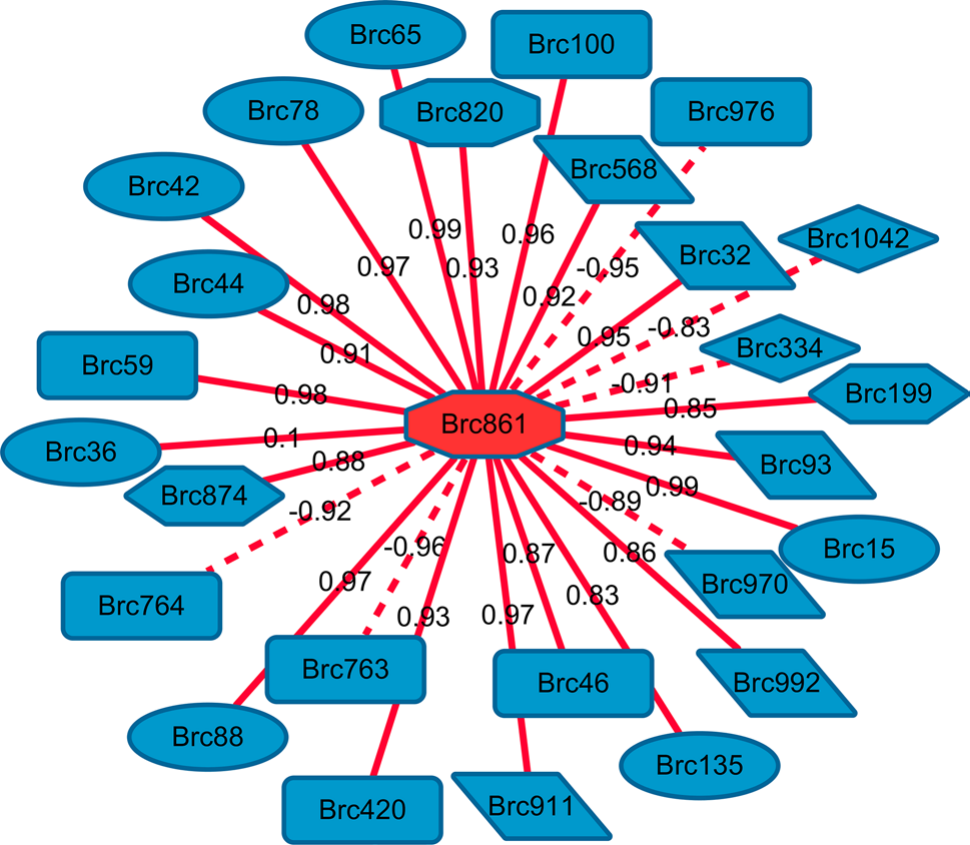
Connection network between glucose 6-phosphate and other differential metabolites. The solid and dashed lines represent positive correlations, respectively, and the number on the line segment represents the correlation coefficient.

We analyzed the correlation between G6P and key DMs and found that 15 of the 111 key DMs were significantly positively correlated with G6P (r > 0.8). These 15 DMs include 6 amino acids, 2 amino acid derivatives, 1 nucleotide derivative, and 5 organic acids and their derivatives (Table S4, Fig. 5). Moreover, six DMs and one nucleotide and their derivatives were significantly negatively correlated with G6P (r <  − 0.8) (Table S4, Fig. 5).

### Analysis of the association between T6P and flowering time genes

**Figure 6:**
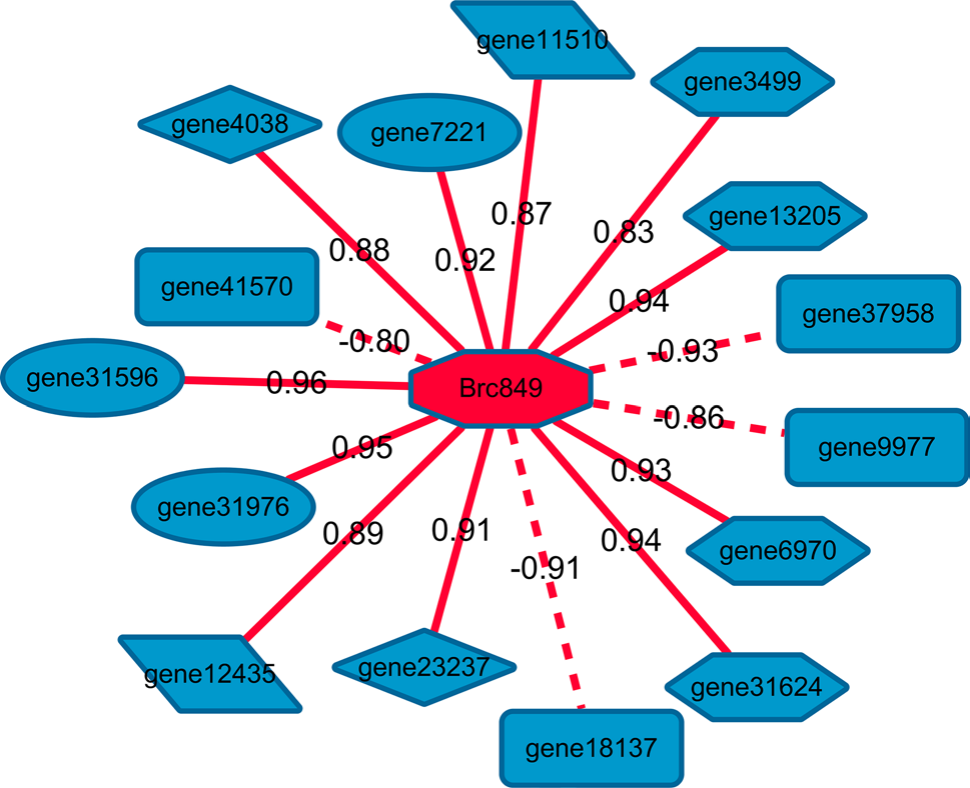
Connection network between trehalose 6-phosphate and the flowering time gene. The solid and dashed lines represent positive correlations, respectively, and the number on the line segment represents the correlation coefficient.

T6P is closely associated with the regulation of plant flowering time^18,22^. During the development of Choy Sum stems, 32 DEGs were involved in the regulation of flowering time (Table S5). The correlation analysis between differential flowering time genes and T6G content indicated a significant positive correlation between three *BcAP1* (gene31596, gene31976, gene7221), three *BcCO* (gene13205, gene31624, gene3499), one *BcFT* (gene6970), two *BcSOC1* (gene11510, gene12435), and two *BcSPL5* (gene23237, gene4038) genes with T6P content, whereas two *BcSVP* (gene18137, gene37958) genes and one *BcTFL1* (gene9977) genes were significantly negatively correlated with T6P content (Table S6, Fig. 6).

## Discussion

Choy Sum, a subspecies of the Chinese cabbage, whose bolted flower stalks are harvested as a food product, has a unique flavor and is rich in nutrients. In the present study, we used wide-target metabolomics to detect and analyze various metabolites at three different stages of flowering in Choy Sum stalk development and correlated the results with existing transcriptome data. Overlapping analysis of the TIC diagrams for the QC samples and multivariate statistical analysis of the metabolites in all samples confirmed that our metabolite research methodology was stable and appropriate, indicating that the data were reliable.

### Metabolomics data shows metabolic fluctuations during Choy Sum stalk development

Previous studies on Choy Sum have been limited to a single metabolite class such as folic acid, vitamin C^23^, phenols^24^, or carotenoids^25^. In this study, we used a widely-targeted metabolomics approach and identified 493 metabolites in 31 categories during the three developmental stages of Choy Sum shoot tips. We found that the shoot tips of Choy Sum contained abundant carbohydrates, amino acids, vitamins, and phenolics; additionally, we identified 26 hydroxycinnamoyl derivatives, 62 flavonoids, and their derivatives.

Using the TCseq package in R, we categorized trends in metabolite changes into four clusters. Most carbohydrates and amino acids increased with Choy Sum stalk development and peaked in S5. However, the levels of most flavonoids, lipids, anthocyanins, and coumarins decreased with the development of Choy Sum stalks. Most vitamins and hydroxycinnamoyl derivatives reached their highest levels at S3. These findings suggest that the Choy Sum stalk is a nutrient sink that accumulates abundant carbohydrates and amino acids during development in preparation for subsequent flowering, fruit, and seed sets.

### Analysis of carbohydrate transport and metabolic regulation in Choy Sum stalks

Source-to-sink carbohydrate transport is a major determinant of plant growth, as it influences numerous physiological processes and traits, such as wheat grain filling^26^, tomato fruit formation^27^, and grape color and yield^28^, across many species. The stalk, the major harvest product of the Choy Sum, is a carbohydrate sink. Carbohydrate accumulation in the stalks determines the quality and flavor of Choy Sum. Our metabolomic analysis indicated that the sucrose content steadily increased during Choy Sum stalk development, which is consistent with a previous report^29^.

Analysis of the differential expression of genes encoding enzymes regulating sucrose biosynthesis revealed that all *BcSS* genes were downregulated and that the initially high expression levels of the *BcSPS* genes decreased as Choy Sum stalk development progressed. These results indicate that sucrose accumulation in the stalk originates mainly from foliar transport and that carbohydrate transport from leaves to storage organs involves phloem loading and unloading as well as carbohydrate transport^30^. Phloem unloading in storage organs entails several transcellular transport steps, including the transfer of sugars from phloem cells to storage cells and the promotion of storage organ growth and/or nutrient accumulation^30–32^, which requires transmembrane carbohydrate transport. Three proteins involved in transmembrane carbohydrate transport have been identified in *Arabidopsis thaliana*, including SUCs^33^, SWEETs^34^, and STPs^35^, all of which participate in two sucrose transport pathways from the phloem to medullary storage cells^36,37^. The first pathway relies on energy-consuming SUC proteins^31^. One study reported that mutations in the *SUC1* gene of maize delays growth and delay flowering, whereas another study indicated that early senescence in sugarcane results from a decrease in the carbon transport capacity^38^. Similar phenotypes were reported to be induced in tomatoes and potatoes by *SUC* knockdown^39,40^. However, in rice, *SUC1* silencing does not induce carbohydrate accumulation or leaf dwarfing, instead, it reduces grain filling^41,42^. Thus, *SUC* regulates phloem loading in source tissues and phloem unloading in sink tissues^43,44^. In the present study, we analyzed the correlation between *SUC* and sucrose accumulation and found that only two genes encoding BcSUC1 (gene32231, gene7395) were significantly positively correlated with sucrose content, indicating that these two genes may be key for transporting sucrose to the shoot tips of Choy Sum.

Our subsequent analyses indicated that glucose and fructose levels increased in the shoot tips during the flowering of Choy Sum stalks. Transcriptome analysis revealed that tonoplast-bound sucrose invertases (*BINV*) were downregulated during Choy Sum stalk development, whereas most cell wall-bound sucrose invertases (*BcCWINV*) were simultaneously upregulated. Thus, glucose and fructose in Choy Sum stalks are transported to the storage cells following extracellular sucrose decomposition. This mechanism is related to the SWEET-dependent energy-independent phloem carbohydrate pathway, wherein sucrose is transported to the vascular parenchyma cells mainly via intercellular filament, and is then actively transported from the vascular parenchyma cells to the apoplast via SWEETs^31^. The SWEET protein has also been detected in the stems of sorghum^30^ and dogtail^45^. The cell wall-bound sucrose invertase CWINV decomposes extracellular sucrose into glucose and fructose, which are transported by SWEET and STP into storage parenchyma cells^30^. One study reported that the antisense expression of the *STP* genes *HT1*, *HT2*, and *HT3* impedes sugar accumulation in tomato fruit parenchyma and reduces yield^46^. Another study indicated that overexpression of the *STP* gene *CsHT3* accelerates metabolism in *Arabidopsis*. Moreover, *CsHT3* upregulation has been reported to induce cucumber fruit enlargement, indicating that cucumber *CsHT3* may play an important role in phloem sucrose unloading^47^.

In the present study, 16 *BcSWEET* and 13 *BcSTP* genes were upregulated during the flowering stage of Choy Sum stalk development. Metabolomic and transcriptome analyses demonstrated that the main carbohydrate accumulation pathways in the Choy Sum stalk include phloem sucrose unloading and transport mediated by BcSWEETs, BcCWINVs, and BcSTPs. Our correlation analysis indicated that 3 *BcCWINV*, 13 *BcSTP*, and t2 *BcSWEET* genes were positively correlated with carbohydrate accumulation, suggesting that sugar transport mediated by the BcSWEET protein might represent the key pathway for sugar accumulation during the flowering of Choy Sum talks. Moreover, BcCWINV can transform extracellular sucrose into glucose and fructose and transport them to storage cells under the action of BcSWEET and BcSTP. This process serves to maintain a low concentration of extracellular sucrose and promotes sucrose in phloem parenchyma cells to be unloaded into the extracellular spaces through BcSWEETs. The expression levels of four sugar transporter proteins, including gene7395 (BcSUC1), gene26037 (BcSWEET1), gene27192 (BcSWEET12like), and gene24551 (BcSTP9) were detected using qRT-PCR, and the results were consistent with the transcriptome data (Fig. S4).

### G6P accumulation is closely related to changes in amino acid metabolites in Choy Sum

Glucose produces G6P under the action of HXK, which then enters the sugar metabolism pathway. We found that during stalk development in Choy Sum, the content of G6P continuously increased. Moreover, two genes encoding HXK (gene134, gene35361) were significantly upregulated and positively correlated with G6P content, indicating their key role in G6P biosynthesis. Under the catalysis of glucose-6-phosphate dehydrogenase, G6P initiates the pentose phosphate pathway to produce 6-phosphoglucoside, which in turn produces 5-phosphate ribose and erythrose-4-phosphate, participating in the biosynthesis of nucleic acids and aromatic amino acids^16,48^. The intermediate of the tricarboxylic acid cycle is the carbon backbone source for synthesizing numerous amino acids, including α-ketoglutaric or oxaloacetic acid. In the present study, the correlation analysis indicated that among the seventeen differentially expressed amino acids and their derivatives, 13 were positively correlated with the G6P content, with 6 amino acids and 2 amino acid derivatives exhibiting a significant positive correlation. We also found that the content of uridine 5′-diphospho-D-glucose was significantly positively correlated with the content of G6P, indicating a close correlation between G6P and the biosynthesis of metabolites, especially amino acids.

### T6P content is closely related to flowering-promoting genes in Choy Sum

The flowering time of plants is influenced by environmental factors such as photosynthesis, temperature, nutrition, and water. Carbohydrates are the main regulatory metabolites during development. In the present study, during the transition from the vegetative to the reproductive stages of plants, sucrose levels in the phloem and stem tips increased. T6P, an intermediate in sugar metabolism, is involved in regulating plant embryonic development, flowering time, meristem determination, and cell fate specificity^17,49^. In *Arabidopsis*, the T6P content increases during the flowering induction period of the stem tip meristem and is closely related to sucrose concentration^50^. Further, downregulation of the T6P synthase gene *TPS1* leads to delayed flowering. Notably, T6P promotes flowering by inducing the expression of SQUAMOSA PROMOTER BINDING PROTEIN-LIKE 3 (SPL3), SPL4, and SPL5^50^. In the present study, the development of Choy Sum stems, the T6P trehalose content continuously increased with the formation of stems and flowers, and the expression levels of the flowering-promoting genes *BcCO*, *BcSOC1*, *BcAP1*, *BcFT*, and *BcSPL5* were directly proportional to T6P content. These findings suggest that T6P may be a signaling molecule that induces the expression of flowering genes during stem development, thereby regulating flowering and bolting in Choy Sum. The expression levels of three flowering-promoting genes, including gene12435 (BcSOC1), gene31976 (BcAP1F), and gene23237 (BcSPL5F) were detected using qRT-PCR, and the results were consistent with the transcriptome data (Fig. S4).

## Conclusions

**Figure 7:**
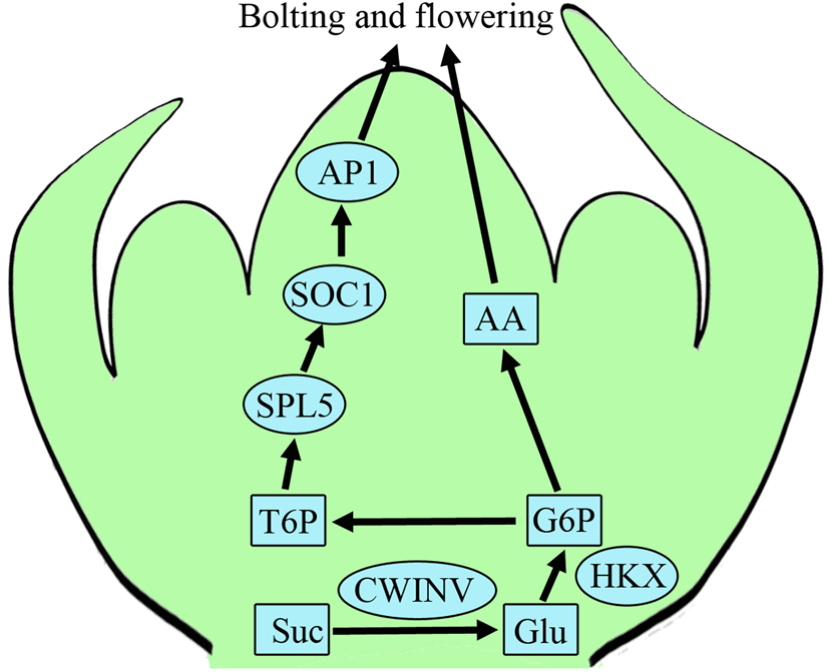
A conceptual diagram of the involvement of carbohydrates in the regulation of flowering and bolting in Choy Sum. Boxes represent metabolites and ellipses represent genes. *Suc* sucrose, *Glu* glucose, *G6P* glucose 6-phosphate, *T6G* trehalose 6-phosphate, *AA* amino acids, *CWINV* cell wall invertases, *HXK* hexokinase, *SPL5* squamosa promoter-binding protein-like5, *SOC1* suppressor of overexpression of CO 1, *AP1* apetala1.

In summary, we applied metabolomics and transcriptomics to investigate changes in metabolite levels in shoot tips during the development of Choy Sum stalks and explore their effects on flowering and bolting. Metabolite analysis indicated that the types and contents of nutrients in stem tips varied with the developmental stage of the stem. We combined metabolomic and transcriptome data to establish a regulatory pathway for the unloading and transportation of carbohydrates from the stem tip phloem to the medullary parenchyma, revealing that G6P is closely related to the synthesis of metabolites such as amino acids. In addition, the analysis indicated a close correlation between T6P and the expression of flower-promoting genes in Choy Sum. Therefore, we speculate that during stalk development, carbohydrates are transported from the leaves to the stem tip through the phloem and are subsequently transported through the thin-walled tissue of the shoot tip via a coordinated interplay involving BCSUC, BcSWEET, and BcSTP. Glucose from the stem tip is catalyzed by HXK to generate G6T, which then forms metabolites, such as amino acids, nucleotides, and lipids. Simultaneously, G6P forms T6P and acts as a signaling molecule to promote the expression of the flowering genes *BcSOC1*, *BcAP1*, *BcFT*, and *BcSPL5*, thereby promoting flowering in Choy Sum (Fig. 7). Nevertheless, further research is required to investigate the effects of changing the content of G6P and T6P in Choy Sum on the content of shoot tip metabolites and flowering. Such investigations will contribute to a deeper understanding of the functions of G6P and T6P in the development of the Choy Sum stalk. The results of this study provide a reference for future research on stalk matter accumulation and the mechanism of flowering regulation in Choy Sum, serving as theoretical guidance for breeding high-quality, high-yield cabbage varieties.

## Methods

### Chemicals and reagents

All chemicals and reagents purchased were of analytical grade and Milli-Q ultrapure water (MerckMillipore, Bedford, MA, USA) was used for all experiments. Methyl alcohol, acetonitrile, and ethyl alcohol were purchased from Merck (Darmstadt, Germany). Authentic standards were purchased from BioBioPha Co., Ltd. (Kunming, Yunnan, China) and Sigma-Aldrich (St. Louis, MO, USA).

### Plant materials

The early-maturing Choy Sum cultivar “Youlv 501 caixin” used in this study was a commercial variety bred by the Guangzhou Institute of Agricultural Sciences (Guangzhou, China). The seeds were purchased from the sales department of Guangzhou Institute of Agricultural Sciences (Guangzhou, China). Plants were grown in a greenhouse (25–30 °C; natural sunlight; sunlight duration was 11–12 h; from late September to early November 2016) at the Guangdong Provincial Engineering Technology Research Center for Protected Horticulture, South China Agricultural University, Guangzhou, China. Choy Sum samples were collected at the S1, S3, and S5 stages (Fig. S5). The stem tips (5 mm) of 50 plants were mixed into one biological replicate, with three biological replicates per stage, flash-frozen in liquid nitrogen, and stored at − 80 °C. Frozen samples were packed on dry ice and delivered to GeneDenovo Inc. (Guangzhou, Guangdong, China) for metabolomic analyses. RNA-seq was performed as previously described, and all sequence data were deposited in the NCBI Sequence Read Archive (SRA, http://www.ncbi.nlm.nih.gov/Traces/sra) under accession numbers SRX2902847 (S1-1), SRX2913033 (S1-2), SRX2913042 (S3-1), SRX2913055 (S3-2), SRX2902847 (S5-1), and SRX2913357 (S5-2)^2^.

### Widely-targeted metabolomic analysis of Choy Sum stalk samples

Metabolite extraction and metabolome analysis were performed according to the methods described by Chen et al.^51^ with some modifications. This metabolite detection method was developed by GeneDenovo Inc. (Guangzhou, Guangdong, China) and has been applied and tested for metabolite analyses in numerous plant species^52,53^.

For the metabolomic analysis, each sample was dried in a freeze-drier and pulverized with zirconia beads for 1.5 min at 30 Hz in a mixer mill (MM 400; Retsch GmbH, Haan, Germany). Subsequently, 100 mg of the ground sample was accurately weighed and 1.0 mL of 70% (v/v) methanol was added to the sample. The suspension was extracted overnight at 4 °C. The extraction efficiency was enhanced by vortexing the suspension three times. The mixture was centrifuged at 10,000×*g* for 10 min (4 °C) and the supernatant was filtered (SCAA-104; 0.22 μm pore; ANPEL, Shanghai, China) before liquid chromatography-tandem mass spectrometry (LC–MS/MS). QC samples were mixed with all samples to confirm the reproducibility of the experiment.

The extracted metabolites were analyzed using an LC–ESI–MS/MS system (UPLC: Shim-pack UFLC; Shimadzu CBM20A; Shimadzu Corp., Kyoto, Japan; MS/MS: Applied Biosystems 4500 QTRAP, Applied Biosystems, Foster City, CA, USA)^17^. A 5 μL aliquot was injected into a Waters ACQUITY UPLC HSS T3 C18 column (2.1 mm × 100 mm; 1.8 μm; Waters Co., Milford, MA, USA) operating at 40 °C and a flow rate of 0.4 mL min^−1^. The mobile phase and elution gradient were established as described by Yang et al.^52^. The effluent was connected to an electrospray ionization (ESI)-triple quadrupole-linear ion trap (Q TRAP)–MS. The API 4500 Q TRAP LC–MS/MS system was fitted with an ESI turbo ion spray interface operating in positive-ion mode and controlled using Analyst v. 1.6 (AB Sciex; Framingham, MA, USA). The ESI source operating parameters were as follows: ESI source temperature, 550 °C; ion spray voltage, 5500 V; curtain gas, 25 psi; and collision-activated dissociation at the maximum setting. QQQ scans were acquired in the form of MRM experiments with an optimized declustering potential and collision energy per individual MRM transition. The m/z range was 50–1000.

Data filtering, peak detection, alignment, and other calculations were performed using Analyst v. 1.6.1 (AB Sciex). Metabolites were identified via internal and public database searches (MassBank, KNApSAcK, HMDB^54^, MoTo DB, and METLIN^55^) and comparison of the m/z, RT, and fragmentation patterns against those for the reference standards.

### Statistical analyses

To compare the various metabolites, PCA and OPLS-DA were performed using the ropls package in R (v. 3.6.2). The OPLS-DA model was corroborated by cross-validation and a 200-permutation test^56^. Significantly discriminant metabolites among the various Choy Sum stalk developmental stages were selected according to the criterion of VIP > 0.7 and were tested for significance at p < 0.05^57,58^. Homogeneous sample clusters identified by metabolomic profiles were used for HCA and heatmap creation, which was carried out in R (v. 3.6.2). Metabolite abundances were log2-normalized. Trends in metabolomic profile changes were determined using the TCseq package in R (v. 3.6.2)^59^.

### Integrative analysis of metabolome and transcriptome data

The transcriptome data were obtained from a previous study^2^, while the transcriptome and metabolome samples were contemporaneous samples. Pearson’s correlation coefficients were calculated to integrate the metabolome and transcriptome data. The mean of all the biological replicates for each cultivar in the metabolome data and the mean value of the expression of each transcript in the transcriptome data were calculated. The fold changes at each developmental stage were then calculated for both the metabolome and transcriptome data. Finally, the omicshare (www.omicshare.com/tools) online platform calculated the correlation between metabolites and gene expression levels, as well as between metabolites. Values within the parameters r > 0.8 or r < − 0.8 and q < 0.05 indicated a significant correlation. Cytoscape (v 3.2.1) was used for visualization^60^.

### qRT-PCR

Four key sugar transporter genes and 3 key flowering genes were selected for RT-PCR detection using *GADPH* as the internal reference gene (Table S7)^61^. Reactions were performed in a LightCycler 480 system (Roche, Basel, Switzerland) with 5 μL SYBR Premix Ex Taq II (Tli RNaseH Plus) (Takara Bio, Dalian, China), 1.5 μL cDNA template, 0.4 μL each primer (10 μmol/μL), and 2.7 μL nuclease-free water. PCR conditions and melting curve analyses were carried out as described by Huang et al.^2^. SigmaPlot v.11 software was used for statistical analysis and data display.

### Research involving plants

The Choy Sum variety used in this study is the “youlv501” variety selected by the Guangzhou Institute of Agricultural Sciences (Guangzhou, China). The seeds were purchased from the sales department of Guangzhou Institute of Agricultural Science and authorized for use, and comply with relevant institutional, national, and international guidelines and legislation.

### Supplementary Information

Supplementary Tables.

Supplementary Figures.

## Data Availability

The data generated or analyzed in this study are included in this published article and its supplementary information file. RNA-seq data were deposited in the NCBI Sequence Read Archive (SRA, http://www.ncbi.nlm.nih.gov/Traces/sra) under accession numbers SRX2902847 (S1-1), SRX2913033 (S1-2), SRX2913042 (S3-1), SRX2913055 (S3-2), SRX2902847 (S5-1), and SRX2913357 (S5-2).
