# Supplementary material for: Widely-targeted metabolomics and transcriptomics identify metabolites associated with flowering regulation of Choy Sum
Source: Sci Rep. 2024 May 9;14:10682. doi: 10.1038/s41598-024-60801-4 (PMC11081954; doi:10.1038/s41598-024-60801-4)
Supplement: Supplementary file 2 — Supplementary Figures. [file 41598_2024_60801_MOESM2_ESM.pdf]

Supplementary Information

Widely-targeted metabolomics and transcriptomics identify metabolites associated with flowering regulation of Choy Sum

Xinmin Huang[1,2], Yunna Zhu[1], Wei Su[1], Shiwei Song[1,*], and Riyuan Chen[1,*]

Supplementary Figures

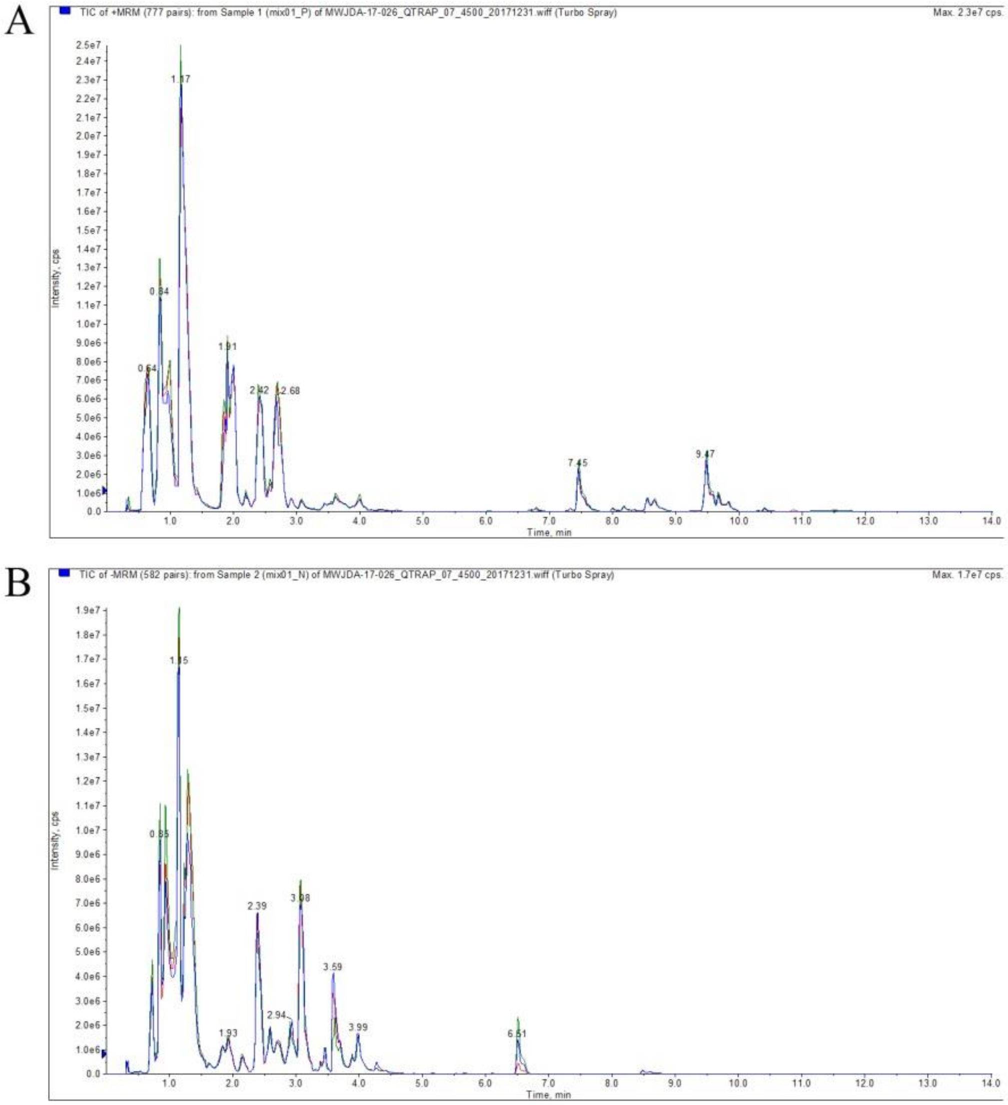

Figure S1. Total ion flow overlap diagram of quality control samples analyzed using mass spectrometry. **A**: Positive ion; **B**: Negative ion**.**

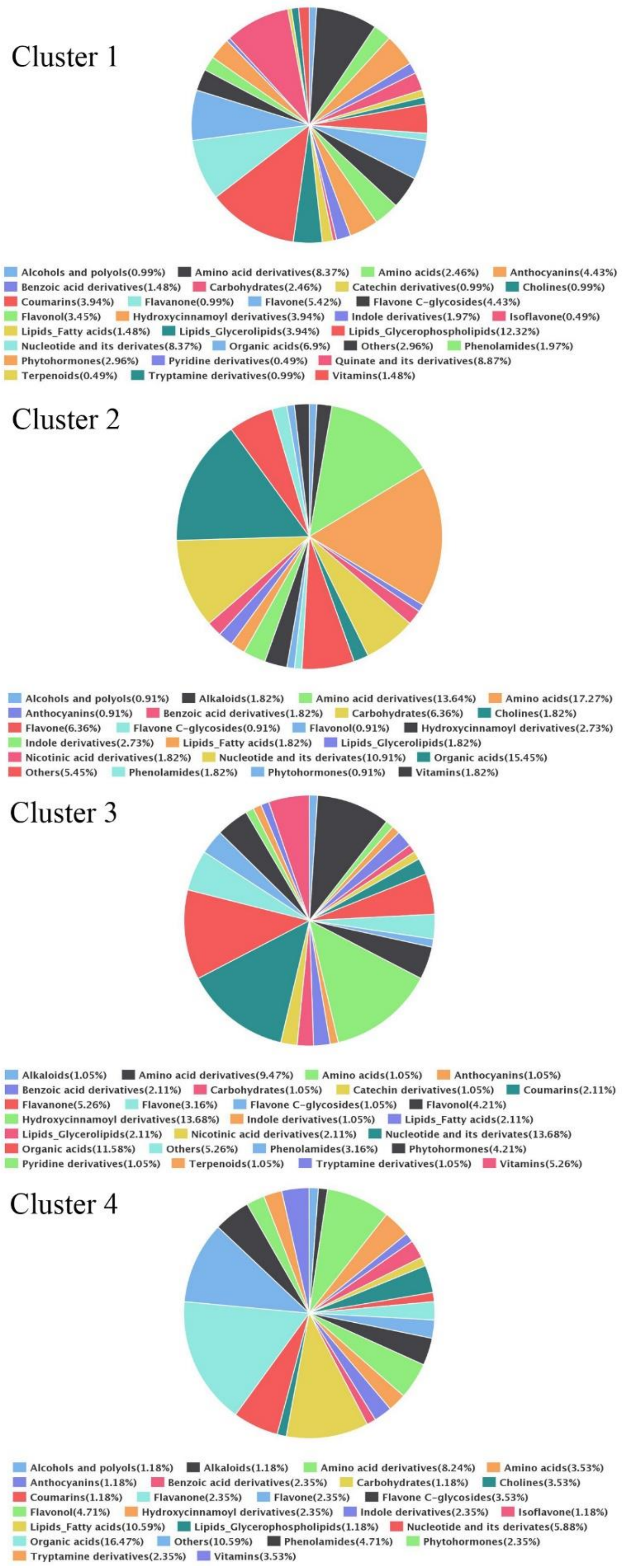

Figure S2. Metabolite distributions in the four clusters

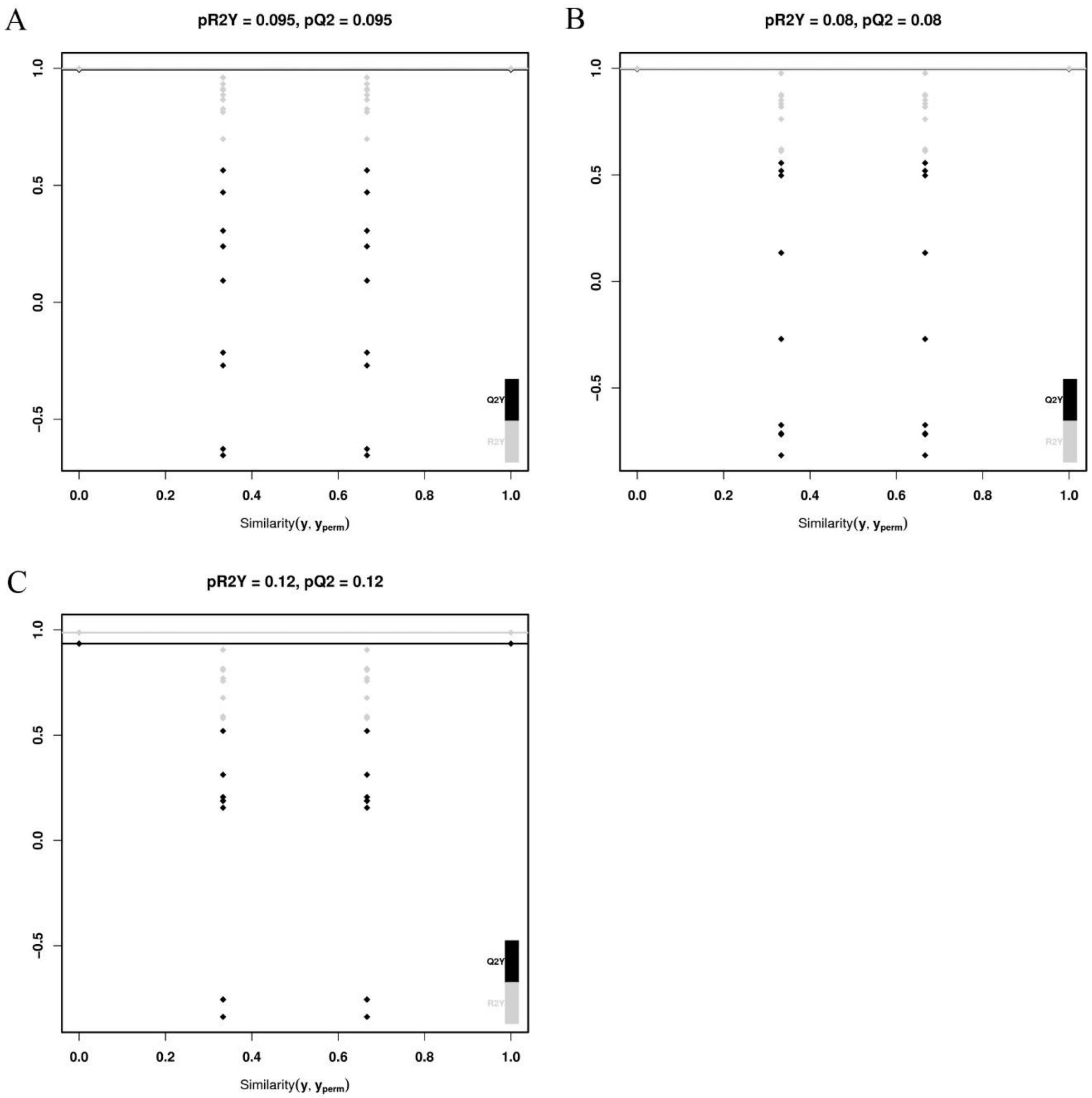

Figure S3. Orthogonal projection to latent structure-discriminant analysis (OPLS-DA) model validation diagram of pairwise comparisons of different Choy Sum stalk developmental stages**.**

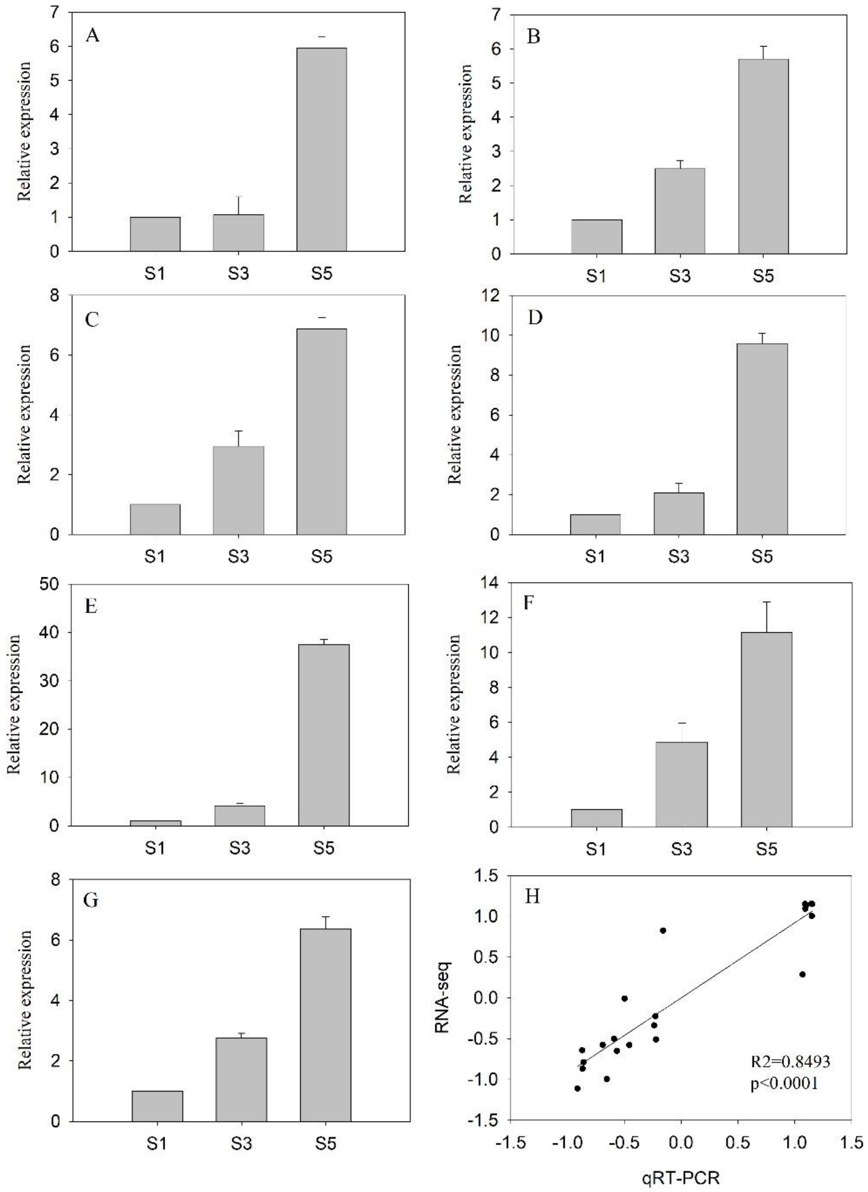

Figure S4. Validation of the expression data from RNA-seq assay using qRT-PCR. A: gene7395 (*BcSUC1*); B: gene26037 (*BcSWEET1*); C: gene27192 (*BcSWEET12like*); D: gene24551 (*BcSTP9*); E: gene12435 (*BcSOC1*); F: gene31976 (*BcAP1F*); G: gene23237 (*BcSPL5F*). H: Pearson's correlations between RNA-seq data and qRT-PCR data used R2 >0.8 as the significance threshold. Values are Z-scores of FPKM and qRT-PCR data.

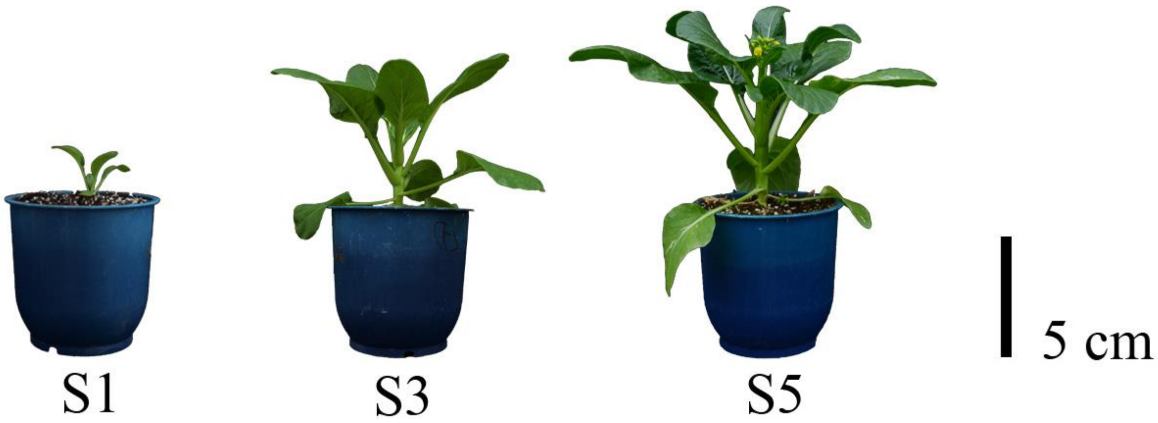

Figure S5. Changes in stalk morphology during the seedling (S1), bolting (S3), and flowering (S5) stages of stalk development. Bar = 5 cm.
